# Remarks on M. Magendie's Late Experiments upon the Nerves

**Published:** 1824-08

**Authors:** John Shaw


THE LONDON

### Medical and Physical Journal.

No 2 of VOL. LII.] AUGUST, 1824. [No 306.

For many fortunate discoveries in medicine, and for the detection of numerous errors, the world is indebted to the rapid circulation of Monthly Journals; and there never existed any work, to which the Faculty, in Europe and America, were under deeper obligations, than to the Medical and Physical Journal of London, now forming a long, but an invaluable, series.—RUSH.

### ORIGINAL COMMUNICATIONS,

### SELECT OBSERVATIONS, &c.

ART. I.—*Remarks on* M. MAGENDIE'S *late Experiments upon the Nerves.* By JOHN SHAW, Esq. &c. &c.

*To the Editors of the Medical and Physical Journal.*

GENTLEMEN,—I feel it due to myself, but more especially to Mr. Bell, to make a few remarks on the questions discussed in the "Appendix" to your Journal of last month.

I shall be obliged to state facts which may seem to bear upon the candour of a gentleman whom I should wish to respect; but, when the merit of such extraordinary discoveries as those which have lately been made on the nervous system is in danger of being frittered away, the truth, however it may operate, must be stated.

The name of M. Magendie is now so well known in this country, that it was not surprising that his late visit to London raised some expectations. There was much interest excited by the performance of the experiments which you have noticed; but, although M. Magendie has been engaged for many years in prosecuting physiology by experiments on living animals, it is extraordinary that the subject which he chose to exhibit in London, as the most interesting and as the most novel, was one which especially belongs to this country, and which had been already fully investigated.

Indeed, I feel myself bound to assert that the principal experiments by which M. Magendie attracted so much attention while in London, were suggested to him by me. I beg, however, to observe, that I had nothing to do with some of the deductions which he drew from them. But I shall go so far as to say, that the inquiry into the functions of the several nerves of the head had never engaged the attention of M. Magendie, until I pointed out to him the differences which there are between them; and especially the dissimilarity of the fifth from the other cerebral nerves.

With a conviction so strong as to induce me to venture on such assertions, I cannot repress my astonishment at the attempts made by M. Magendie's countrymen, and even by a few of our own, to give him credit for discoveries which I am still willing to believe he would not take to himself.

I shall now state the grounds on which I consider myself warranted in making the above assertions.

While in Paris, in the autumn of 1821, I was introduced to M. Magendie. Mr. Bell's discoveries on the nervous system naturally became the subject of conversation; and M. Magendie was so much interested by the facts which I stated, that he begged me to show him some of the experiments in proof of them. For this purpose we went out, between five and six in the morning, to the Veterinary College of Alfort, about five miles from Paris; and there, in presence of the Professor of Alfort, of Dr. Spurzheim, and a number of French gentlemen, I performed several experiments, (and among others that of dividing the fifth pair,) in example of the principles on which Mr. Bell had founded his opinions of the nervous system. At the same time, I presented to M. Magendie a short account of the system, (as it still stands,) and copies of the two plates which are given in the Medical Journal for December 1822 and June 1823. These plates, your readers may recollect, not only illustrate the difference between the two grand classes of nerves, but also show the similarity between the fifth and the spinal nerves.

In less than a month after this, the French Journals were full of Mr. Bell's discoveries, and, as I had expected, they were immediately taken up warmly in this country.

But the avidity with which every thing foreign is now received here, has made it necessary for me to come forward more than once to prevent the information, which came second-hand from Paris, being considered original.

This has been rather a difficult task; for, in consequence of several claims having been permitted to remain some time without being challenged, the assertions of the French in favour of their countryman have, even by some of our friends, been considered justly founded. I therefore hoped that M. Magendie's arrival in this country would be so far useful to the subject, as to excite attention to facts which have been hitherto little attended to, except by those who have been pupils in the school of Great Windmill-street: and it was exactly as I expected; for M. Magendie was invited to make his experiments, and the results were so striking, that the mass of a large assemblage of spectators were so much delighted as to shake hands with each other, and congratulate themselves on having witnessed these extraordinary and *novel* facts. However, a few,—although I

fear, from what I saw, (for I myself was present,) but a very few,—were as much astonished that it did not seem to be known that the experiments, which were exciting so much wonder, had been repeatedly done in the rooms on the other side of the street; and that, years ago, accounts of them had been published in all sorts of Journals, English and foreign.

I shall now ask, whether the slightest hint of the present views of the nervous system is to be found in any of M. Magendie's works, previously to the autumn of 1821,—that is, before I informed him of the discoveries for which many are still ready to give him credit. That I first pointed out to him the difference of the functions of the several nerves, does not rest on my assertion alone; for the first notice of the subject, to be found in his works, is in his Journal for October 1821, and is a quotation from the account which I gave him. I trust this will be sufficient to prove that, in the investigation of the functions of the several nerves, M. Magendie has not the merit of originality; and that the experiments which he has since made on this question are only following up Mr. Bell's inquiries.

Perhaps, it may still be said that M. Magendie has made some discoveries without having received any previous hints, as M. Cuvier ascribes to him the merit of having discovered the difference of the functions of the two roots of the spinal nerves.* But if, after the proofs which were offered in the Number of this Journal for October 1822, this idea still holds, I shall despair of being able to convince your readers to whom the merit of having discovered the functions of the fifth pair belongs. I ought, however, in justice to acknowledge, that I have no reason to suppose that Mr. Bell's claims to being the discoverer of the difference in the functions of the roots of the spinal nerves, is now disputed by any Englishman. Indeed, in all the works which have been lately published, it is fully admitted.

After reading the papers of M. Magendie, and the remarks which you have made upon them, one scarcely knows whether to be more surprised at the apparent attempt to give as a newly discovered fact, that the fifth pair is the nerve which bestows sensibility on the principal parts of the head, or at the singularly inconclusive reasoning and fallacy of the deductions from the experiments beyond what we already know. This is strong

---

* See the *Rapport sur l'Etat de l'Histoire Naturelle et sur les Accroissements depuis le Retour de la Paix Maritime.* Par M. le Baron Cuvier.

M. Cuvier's remarks upon the progress of physiology in France appear curious, when compared with the observations which you have made at pages 10 and 15 of your Retrospect, on the contradictory statements of the results of similar experiments made by Flourens, Serres, Magendie, Rolando, Fodera, and Bailly.—"La rigueur des experiences auxquelles on l'a soumise, a imprimé a la science, qui en traite une caractere de precision, [it is of the experiments on the brain he speaks!] dont a peine il y a cinquante ans l'aurait on crue susceptible."

language; but surely it is excusable, and even necessary, when the attempt to overturn opinions which have been held for ages, and conclusions which have been drawn from a patient investigation of the anatomy of man and of the lower animals, and the results of experiments founded on these investigations, rests on no firmer basis than on experiments made at random, from which the author conceives, although he has not the slightest proof, that he has shown that the olfactory nerves are not for smell,—that the optic nerves are not for vision,—and that the auditories are not for hearing; but that all these fine and curious senses are supplied by a nerve, which at the same time gives the power of motion to the muscles of the jaws, and common sensibility to the surfaces of the head. It was really a bold conclusion to come to, that the results of such experiments were sufficient to prove the necessity of the general theory of sensations being reformed. "Si ce dernier resultat est exact, tous les sens seraient donc sous l'influence de la cinquieme paire, et la theorie generale des sensations devrait donc etre reformée."

Although it is scarcely necessary, after the arguments which you have offered in contradiction of M. Magendie's views, to give any further illustrations to prove the complete error into which he has fallen, not only regarding the functions of the olfactory nerve and of the fifth, but even of the most common phenomena of breathing and smelling, I may remark that I might have stated, many years ago, that the portio dura is the nerve of smell, with as much reason as M. Magendie now believes the fifth to be so; for I could offer as strong proofs on the one side as the other. After cutting the right or left portio dura, the animal no longer smells by the same nostril, nor is it even irritated if ammonia be held under the nostril, although the fumes are so strong as to make his eye water. Indeed, even in cases where the portio dura of one side is paralysed, the patient no longer smells with the corresponding nostril. Has M. Magendie's experiments shown more than this, or even so much? By cutting the fifth pair, he rendered the surfaces of the nose insensible to the irritation of the caustic fumes; but have we any proof that the animal could not *smell?* I believe that, if we destroy the sensibility of the membrane, we shall very much impair the organ; but still these experiments afford no proof that the *sense of smell* is destroyed.

Indeed, no better example can be afforded of the justness of the remarks in your Historical Retrospect, on the manner in which experiments are now conducted in France, and of the mistakes into which we may be led by attaching importance to experiments which are made at random, and without a previous careful consideration of the structure and functions of each part. Had M. Magendie recollected that the nose was a complicated organ, requiring several distinct nerves, he would have at once seen that its functions could not be properly performed, if any one of its parts were destroyed. Thus, if the portio dura be cut, the power of inhaling, in that particular manner which is necessary to smelling, is lost; and hence the sense is impaired.

With regard to the question of the influence of the fifth over the functions of the eye, I shall presently make a few observations; but, before doing so, I shall offer proofs, which I trust will be satisfactory to the most sceptical, that M. Magendie has not the slightest claim to the merit of having discovered any one of the functions of the fifth nerve.

I believe that the general impression among those who have interested themselves in this question is, that, with the exception of supposing the fifth to be the nerve of smell, sight, and hearing, (an idea, of which there is no desire to rob its author,) all the facts regarding the functions of the fifth had been previously described by Mr. Bell, or by myself.

In alluding to these papers on the sensibility of the parts about the head, and especially on the functions of the fifth pair, you have stated, that "M. Magendie really writes and speaks as if he had not read the papers containing these researches." It is not surprising that those who read his papers, or who heard his remarks, or who knows the manner in which he has since distributed his papers in London, should form this opinion. I was never more astonished than (when standing by M. Magendie on the occasion you mention,) to hear him state as a new fact, that the fifth pair was the nerve for the sensibility of the parts about the head,—that it conveyed, or continued, the sensibility from the spinal marrow to the head; and, that he wished to impress this upon his hearers as a *novel* fact was inferred, not only by his making the experiments to prove it, but also by his begging a gentleman near him to translate the above information for the benefit of those of his audience who did not understand French. I affirm, that at the moment I could not help believing that he had not read any of the numerous papers which had been published, or that he had forgotten all which I had told him about the fifth pair, when in Paris. With this impression I went home to examine his own Journals. I have made the following extracts from them, *being translations from papers which I had transmitted to him*. To those who may imagine that any of the functions of the fifth were discovered by M. Magendie, these extracts will afford sufficient proofs that, although injudicious friends may still endeavour to support his claim, he could scarcely have intended to take this merit to himself, notwithstanding the contents of his last papers.

"Le degré de sensibilité dans les deux joues était exactement le même. Cette seule circonstance suffisait pour être sur que le nerf de la cinquième paire, était intact a tous égards mêmes dans les parties les plus inferieures de la face du côte droit."—138. (*Avril* 1822.)

"Pour constater l'integrité des branches de la cinquième paire dans le nez, je touchai l'interieur de la narine droite avec un plume, et aussitot des signes d'eternuement se manifesterent sur le côte opposé."—139.

These extracts are from the description of an instance of paralysis of the face, in which the portio dura only was affected, and were offered to prove that, since the sensibility of the parts was entire, the fifth nerve must still be perfect.

The following are from the description of a case of hemiplegia, and were given to prove that the fifth nerve of one side was affected.

"J'examinai après le degré de sensibilité dans les deux joues.

"On piqua la joue droite avec une aiguille, il resta parfaitement tranquille quoique le sang en sortit. On voulut faire cette operation du coté gauche, mais il retira la tête tout a coup et temoigna de la douleur. On observait la même difference de sensibilité en tirant un poil de sa barbe du chaque coté."

"On s'assura de l'état de la cinquième paire à l'intérieur de nez. En chatouillant la narine droite aucun effet ne fut produit, mais en chatouillant la narine gauche l'éternument s'effectua."

I have not thought it necessary to give the translation of these extracts, as the original papers may be found in the Journal of Science, and copious similar passages in the Philosophical Transactions; one of which I give from Mr. Bell's papers:

"The fifth is the universal nerve of sensation to the head and face, to the skin, to the surfaces of the eye, the cavities of the nose, mouth, and tongue."

I shall make only one other extract, which will be a conclusive proof that, in all the investigations into the functions of the fifth, its influence over the schneiderian membrane was considered an important part of the inquiry. After having, in the description of a case where both the portio dura and the fifth were injured, shown that one cheek and the corresponding side of the tongue were insensible, I made the following remark; and which, as it was copied into M. Magendie's Journal, I shall give in French:—"Nous oubliames de constater le degré de sensibilité de l'interieur de la narine, aussi sommes nous tout a fait incapables d'assurer si les divisions de la cinquième paire qui se rendent dans ces parties étaient paralysées."

To make my position still stronger, I may repeat, that, while in Paris in 1821, I divided the portio dura and the fifth in a horse; and that a notice of the results of this experiment is given in M. Magendie's Journal for January 1822.

In the remarks on M. Magendie's papers, you have fallen into a slight mistake, in supposing that we had not experimented on the fifth, except upon the face; but that this had been first done by M. Magendie. By referring to the Medical and Physical Journal for October 1822, you will find that I had cut the nerve of an ass close to the brain, to show the effects of its division on the temporal and masseter muscles; and in the same paper it is also stated, that I irritated the nerve while in the spheno palatine fissure, to further prove the influence which it has, as a double nerve, over the muscles and the skin. In the Philosophical Transactions for 1823,* the following remarks are made by Mr. Bell:—" I have not been able to excite the motions of the eye by irritating the ophthalmic division of the fifth after the division of its root;" and in the same volume, in a note, he adds—" In attempting to excite the muscles of the eye by galvanism sent through the fifth nerve, the muscles of the jaw were affected."

Although the effects produced by cutting the nerves after their exit from the skull have more especially interested us, the circumstances just stated will show that the functions of every part of the fifth pair have been fully investigated. This will still be more clearly shown by the remarks which I shall take the liberty of adding to those you have already made, in criticising M. Magendie's views of the influence which the fifth has over the organ of vision.

The supposition of M. Magendie, that the fifth, and not the optic, is the nerve of vision, is founded on such slight grounds, that it scarcely requires serious refutation. Had an animal continued to see after the optic nerve was cut through, we might have been led to believe that there was no trick nor deception practised in the case of the famous Miss M'Avoy, of Liverpool, and that this young lady really could see with the tips of her fingers, as she pretended to do; because we know that the spinal nerves have been proved to be in every respect similar to the fifth, (M. Magendie's new nerve of vision.) But the truth is, that, although the fifth be paralysed, the vision is not destroyed; it is only impaired in a manner which can be easily understood. The following cases will not only prove

* " I cut a branch of the fifth upon the face; the sensibility of the corresponding side of the lip was destroyed, but little paralysis ensued, except of certain actions of the orbicularis oris. I cut the nerve nearer the brain, and at a point previous to its having given off the branches to the other muscles; then the jaw fell, and the muscles of that side were powerless. I varied the experiment by irritating the nerve where it lies in the spheno palatine fissure, immediately after an animal was killed. The jaws then came together with much force; indeed, so as to nip my assistant's finger severely. This last experiment may be compared with the very common one of galvanizing the nerves which pass from the spinal marrow to supply the muscles of the extremities."

this, but also that all the interesting circumstances regarding the eye have been previously described, either by Mr. Bell or by me. The extract in which the question of the effect of the fifth over the motions of the iris is considered, and in which it is also shown that, although the fifth was destroyed, the patient could see, was published by me in the Medico-Chirurgical Transactions for 1822.

"No explanation has as yet been given why the pupil should, in some cases of amaurosis, remain fixed and unaltered by the exposure of the eye to the different degrees of light, while in others it is as sensible to the stimulus of light as a perfect eye. It will be very difficult to obtain any conclusive evidence on this question; but circumstances seem to prove thus far, that the motions of the iris depend upon other causes than merely the state of the retina. They are perhaps, in a great measure, controlled by the fifth nerve. In support of this opinion, which however I offer as a mere speculation, I may adduce the common occurrence of a slight paralysis of the levator palpebræ, and of the iris, without the retina being at the same time affected. We know that the fifth pair supplies the levator palpebræ, and that the ciliary nerves which go to the iris are connected with it.

"A good example of complete paralysis of the levator palpebræ, and of loss of power in the pupil, without any affection of the retina, occurred last winter; and I was fortunate enough to have an opportunity of examining the body after death. A young woman had a fungous tumor under the jaw; the cheek of the same side was paralytic; the upper eyelid of the same side had fallen; but, when the eyelid was raised, the patient could see distinctly, although the pupil was fully dilated and immoveable. Upon dissection, it was found that the tumor had extended into the lateral part of the orbit; the fourth nerve ran over the tumor, the third was in the substance of it, but the ophthalmic division of the fifth pair was the nerve most destroyed; the sixth was partially affected. The tumor did not reach as far as the optic nerve. Since all the nerves of the orbit, except the optic, were included in the disease, we cannot draw any further conclusion from this case, than that the motions of the iris do not altogether depend upon the state of the optic nerve. The voluntary power which some individuals possess over the motions of the iris, will perhaps be considered as in some degree supporting the view which I have taken. The members of the Society are, no doubt, aware that one of their most distinguished associates has this voluntary power over the motion of his iris. Upon an occasion in which the gentleman I allude to, was so kind as to show me to what an extent he could exercise this power, I thought I could perceive that the exertion which attended the attempt had some effect upon the motions of the upper eyelid."

In another case, which is noted in Mr. Bell's paper read before the Royal Society in March 1823, where there were indubitable proofs of the paralysis of the fifth pair, the patient could see; and this is proved in a curious way: for, so far was he from being blind, although the eye was insensible to touch, and the cornea even slightly opaque, he could perceive a red light even when the eyelid was drawn down. Some of your readers may remember the explanation of this—that, unless the eye turns up at the time the eyelids are closed, there is not complete darkness: this patient had lost all power of moving the eye.*

You have already mentioned the case which was transmitted by Mr. Crampton, of Dublin, to Mr. Bell. In this instance, also, although the ophthalmic division of the eye was paralysed, the young lady could see distinctly. With regard to the other phenomena which ensue when the fifth is destroyed, in the manner described by M. Magendie, I agree with you that nothing can be deduced from them; for I hold it to be almost impossible to destroy the fifth, in the manner done by M. Magendie, without injuring, more or less, some of the other nerves. It would, indeed, be very extraordinary if, after such an operation behind the orbit, and after depriving the animal of the power of closing the eye, a violent inflammation, such as is described, had not been excited. But, even in M. Magendie's reasoning upon this part of the subject, we have another proof of the justness of the observations in your Historical Retrospect upon the present system of making experiments. Thus, by the remarks which M. Magendie has made upon the animal not winking after the sensibility of the cornea is destroyed, he shows that he is unacquainted with one use of the eyelids, of which he could scarcely have been ignorant had he studied the comparative anatomy of the eye. Again, in drawing the comparison between the consequences of merely paralysing the eyelids by cutting the portio dura, and of rendering the eye senseless and motionless by his operation behind the orbit, he seems not to have known that, by his last experiment, he not only deprived the eyelids of the excitement to wink, but, by depriving the eyeball of motion, he put a stop to the movements of the haw, which forms a most essential part in the economy of the eyes of quadrupeds, being in fact a third eyelid, and which is not at all affected by cutting the portio dura. (See a paper in the Philosophical Transactions of 1823, by Mr. Bell.)

---

* I have seen this patient within the last ten days: his case, which is very curious and interesting, will be given in Mr. Bell's Work upon the Nerves.

I have thus, I trust satisfactorily, established the claims of Mr. Bell, to the discovery of the functions of the fifth pair of nerves; a duty which was incumbent on me to perform, as it was through me that the facts were obtained which have been made the grounds of a claim on the part of another. But I am happy to think that this is the last occasion on which there will be any necessity for such discussions, as, in the course of a few weeks, Mr. Bell will publish a connected account of his discoveries, with plans and illustrations.

*London; July 3, 1824.*

