# Errata in Our Last Number

**Published:** 1824-09

**Authors:** 


					Errata in our last Number.
In Mr.Shaw's Paper, page 103, line 5 from the bottom, for hair read haw.

				

